# Score-based tests of measurement invariance: use in practice

**DOI:** 10.3389/fpsyg.2014.00438

**Published:** 2014-05-30

**Authors:** Ting Wang, Edgar C. Merkle, Achim Zeileis

**Affiliations:** ^1^Department of Psychological Sciences, University of MissouriColumbia, MO, USA; ^2^Department of Statistics, Faculty of Economics and Statistics, Universität InnsbruckInnsbruck, Austria

**Keywords:** measurement invariance, factor analysis, lavaan, parameter stability, ordinal variable, structural equation modeling

## Abstract

In this paper, we consider a family of recently-proposed measurement invariance tests that are based on the *scores* of a fitted model. This family can be used to test for measurement invariance w.r.t. a continuous auxiliary variable, without pre-specification of subgroups. Moreover, the family can be used when one wishes to test for measurement invariance w.r.t. an ordinal auxiliary variable, yielding test statistics that are sensitive to violations that are monotonically related to the ordinal variable (and less sensitive to non-monotonic violations). The paper is specifically aimed at potential users of the tests who may wish to know (1) how the tests can be employed for their data, and (2) whether the tests can accurately identify specific models parameters that violate measurement invariance (possibly in the presence of model misspecification). After providing an overview of the tests, we illustrate their general use via the R packages *lavaan* and *strucchange*. We then describe two novel simulations that provide evidence of the tests' practical abilities. As a whole, the paper provides researchers with the tools and knowledge needed to apply these tests to general measurement invariance scenarios.

## 1. Introduction

Some of the papers in this special issue focus on the topic of approximate measurement invariance: we know that strict hypotheses of measurement invariance do not hold exactly across different groups, and this should be reflected in corresponding tests of measurement invariance. Under a Bayesian approach, we may implement the idea of approximate invariance (e.g., Muthén and Asparouhov, 2013) by replacing across-group equality constraints on parameters with informative prior distributions. In this paper, we describe an alternative approach: the development of test statistics that are especially sensitive to violations that are monotonic w.r.t. the variable of interest (and less sensitive to violations that are non-monotonic w.r.t. the variable of interest). Because monotonic violations are more likely to be interpretable and interesting to the researcher, we can gain more power to detect these violations by de-emphasizing other types of violations. The resulting test statistics are specifically applicable to situations where one wishes to test for measurement invariance with respect to an ordinal variable, and they are special cases of a family of tests that may be used to study measurement invariance w.r.t. continuous, categorical, and ordinal variables.

The study of measurement invariance w.r.t. categorical auxiliary variables (via, e.g., likelihood ratio tests) is popular and well known, and ordinal auxiliary variables are typically treated as categorical in measurement invariance contexts. The study of measurement invariance w.r.t. continuous variables is newer and less known: along with the family described here, other methods include moderated factor models (Purcell, 2002; Bauer and Hussong, 2009; Molenaar et al., 2010) and factor mixture models (Dolan and van der Maas, 1998; Lubke and Muthén, 2005). These methods require estimation of a model of greater complexity, while the tests described in this paper work solely on the output of a traditional factor model (see Merkle and Zeileis, 2013, for further comparison of these methods). These methods all assume that the estimated model is correctly specified, save possibly for differences in parameter values between individuals.

The family of tests described here have recently been applied to the study of measurement invariance (Merkle and Zeileis, 2013; Merkle et al., 2014), though their practical application has been limited to a small set of simulations and data examples. In this paper, we provide a detailed illustration of the tests' use and performance under scenarios likely to be encountered in practice. While the previous papers have described and studied the tests under ideal conditions, we focus here on two topics of interest to the applied researcher: software considerations for carrying out the tests, and test performance under model misspecification. The latter issue is particularly important because, in practice, all models are misspecified. Hence, practically-useful tests of measurement invariance should be robust to model misspecification.

In the following sections, we first briefly review the theoretical framework of the proposed tests and provide a short tutorial illustrating the use of the tests in R (R Core Team, 2013). Next, we study the tests' performance in simulations that mimic practical research scenarios. Finally, we provide some further discussion on the tests' use in practice.

## 2. Background

This section contains background and discussion of the proposed statistics as applied to structural equation models (SEMs); for a more detailed account, see Merkle and Zeileis (2013) and Merkle et al. (2014). For details on the statistics' application to general statistical models, see Zeileis and Hornik (2007).

As currently implemented for SEM, the statistical tests described in this paper can be applied to models that are estimated via a multivariate normal or Wishart likelihood (or discrepancy) function, with extension to other discrepancy functions being conceptually straightforward. The tests are carried out following model estimation, making use of output associated with the fitted model. In general, we fit a model that restricts parameters to be equal across observations, then carry out a *post hoc* test to examine whether specific parameters vary across observations. This procedure is similar in spirit to the calculation of modification indices (Bentler, 1990) and to Lagrange multiplier tests (Satorra, 1989), and, in fact, those statistics can be viewed as special cases of the family described here.

Following model estimation, the tests primarily work on the *scores* of the fitted model; these are defined as

(1) $$s(\theta ;x_{i})={\left(\frac{\partial \ell (\theta ;x_{i})}{\partial \theta_{1}},\ldots ,\frac{\partial \ell (\theta ;x_{i})}{\partial \theta_{k}}\right)}^{⊤}\text{​},\;i=1,\ldots ,n,$$

where ℓ(θ; *x*_*i*_) is the likelihood associated with individual *i* and θ is a *k*-dimensional parameter vector. The corresponding maximum likelihood estimate $\hat{\theta }$ solves the first order condition: $\sum_{i\;=\;1}^{n}s(\hat{\theta };x_{i})=0$.

To verbally describe Equation (1), each individual has *k* scores describing the extent to which the fitted model describes that particular individual. These scores are similar to residuals and, in fact, the tests can be applied directly to residuals in other contexts (see Zeileis and Hornik, 2007): we can roughly interpret scores near zero as providing a “good” description of an individual, with scores far from zero providing a “bad” description of an individual. This is only a rough interpretation as, even when measurement invariance holds, some individuals' scores will be further from zero than others. However, under measurement non-invariance, the scores will differ for different subgroups of individuals (say, scores in subgroup A tend to be negative and scores in subgroup B tend to be positive). Each of the *k* scores represents one model parameter, which, as further described below, allows us to test subsets of model parameters for invariance. While scores can be obtained under the multivariate normal likelihood (discrepancy) function and alternatives such as generalized least squares, most SEM software fails to supply the scores to the user.

To use the scores for testing, we order individuals according to an auxiliary variable *V* (the variable against which we are testing measurement invariance) and look for “trends” in the scores. For example, imagine that we are testing for measurement invariance w.r.t. age. If there exists measurement non-invariance w.r.t. age, then some parameter estimates may be too large for young individuals and too small for old individuals (say). This result would be reflected in the scores, where young individuals' scores may be greater than zero and old individuals' scores less than zero (though the sign of the scores will depend on whether a function is being minimized or maximized). Conversely, if measurement invariance holds, then all individuals' scores will fluctuate randomly around zero.

To formalize these ideas, we define a suitably scaled cumulative sum of the ordered scores. This may be written as

(2) $$B(t;\hat{\theta })\;=\;{\hat{I}}^{-1/2}n^{-1/2}\sum_{i\;=\;1}^{\lfloor n\cdot t\rfloor }s(\hat{\theta };x_{\left(i\right)})\;(0\leq t\leq 1)$$

where ***Î*** is an estimate of the information matrix, ⌊*nt*⌋ is the integer part of *nt* (i.e., a floor operator), and *x*_(*i*)_ reflects the individual with the *i*-th smallest value of the auxiliary variable *V*. While the above equation is written in general form, we can restrict the value of *t* in finite samples to the set {0, 1/*n*, 2/*n*, 3/*n*, …, *n/n*}. We focus on how the cumulative sum fluctuates as more individuals' scores are added to it, e.g., starting with the youngest and ending with the oldest individual if age is the auxiliary variable of interest. The summation is premultiplied by an estimate of the inverse square root of the information matrix, which serves to decorrelate the fluctuation processes associated with individual model parameters while preserving the behavior of individual parameters' fluctuations.

Under the hypothesis of measurement invariance, a central limit theorem can be used to show that the fluctuation of the above cumulative sum follows a Brownian bridge (Hjort and Koning, 2002). This result allows us to calculate *p*-values and critical values for test statistics under the hypothesis of measurement invariance. We can obtain test statistics associated with all model parameters and with subsets of model parameters.

Multiple test statistics are available, depending on how one summarizes the behavior of the cumulative sum of scores. For example, one could take the absolute maximum that the cumulative sum attains for any parameter of interest, resulting in a *double max* statistic (the maximum is taken across parameters and individuals). Alternatively, one could sum the (squared) cumulative sum across parameters of interest and take the maximum or the average across individuals, resulting in a *maximum Lagrange multiplier* statistic and *Cramér-von Mises* statistic, respectively (see Merkle and Zeileis, 2013, for further discussion). These statistics are given by

(3) $$DM=\underset{i\;=\;1,\ldots ,n}{\max}\underset{j\;=\;1,\ldots ,k}{\max}|B{\left(\hat{\theta }\right)}_{ij}|$$

(4) $$CvM=n^{-1}\sum_{i\;=\;1,\ldots ,n}\sum_{j\;=\;1,\ldots ,k}B{\left(\hat{\theta }\right)}_{ij}^{2},$$

(5) $$\max LM=\underset{i\;=\;\underline{i},\ldots ,\bar{ı}}{\max}\;{\left\{\frac{i}{n}\left(1-\frac{i}{n}\right)\right\}}^{-1}\sum_{j\;=\;1,\ldots ,k}B{\left(\hat{\theta }\right)}_{ij}^{2}.$$

Critical values associated with *DM* can be obtained analytically, while critical values associated with the other statistics can be obtained from direct simulation (Zeileis, 2006) or from more refined techniques (Hansen, 1997). This issue should not be important to the user, as critical values are obtained directly from the R implementation described later.

Importantly, the above statistics were derived for situations where individuals are uniquely ordered according to the auxiliary variable. This is not always the case for measurement invariance applications, where the auxiliary variable is often ordinal. To remedy this situation, Merkle et al. (2014) extended the framework to situations where one has an ordinal auxiliary variable of interest. Essentially, one allows all individuals with the same value of the auxiliary variable to enter into the cumulative sum at the same time. Analogous test statistics are then computed, with modified critical values being adopted to reflect the change in the statistics' computation.

For an ordinal auxiliary variable with *m* levels, these modifications are based on *t*_ℓ_ (ℓ = 1, …, *m* − 1), which are the empirical, cumulative proportions of individuals observed at the first *m* − 1 levels. The modified statistics are then given by

(6) $$WDM_{o}=\underset{i\in \{i_{1},\ldots ,i_{m-1}\}}{\max}\;{\left\{\frac{i}{n}\left(1-\frac{i}{n}\right)\right\}}^{-1/2}\underset{j\;=\;1,\ldots ,k}{\max}|B{\left(\hat{\theta }\right)}_{ij}|,\;$$

(7) $$\max LM_{o}=\underset{i\in \{i_{1},\ldots ,i_{m-1}\}}{\max}\;{\left\{\frac{i}{n}\left(1-\frac{i}{n}\right)\right\}}^{-1}\sum_{j=1,\ldots ,k}B{\left(\hat{\theta }\right)}_{ij}^{2},$$

where *i*_ℓ_ = ⌊*n*· *t*_ℓ_⌋ (ℓ = 1, …, *m* − 1). Critical values associated with the *WDM*_*o*_ statistic can be obtained directly from a multivariate normal distribution (see Hothorn and Zeileis, 2008), while critical values associated with max *LM*_*o*_ can be obtained via simulation. This simulation is somewhat computationally intensive and, in practice, takes about 10 min on the authors' computers when 50,000 replications are sampled from the approximate asymptotic distribution. However, the wait is often worth it, as Merkle et al. (2014) found the performance of the max *LM*_*o*_ statistic to have more power than the *WDM*_*o*_ statistic and the traditional likelihood ratio test statistic when the measurement invariance violation is monotonic with the ordinal variable.

Finally, if the auxiliary variable *V* is only nominal/categorical, the cumulative sums of scores can be used to obtain a Lagrange multiplier statistic. This test statistic can be formally written as

(8) $$LM_{uo}=\sum_{\ell =1,\ldots ,m}\sum_{j=1,\ldots ,k}{\left(B{\left(\hat{\theta }\right)}_{i_{\ell }j}-B{\left(\hat{\theta }\right)}_{i_{\ell -1}j}\right)}^{2}\text{​},$$

where $B{\left(\hat{\theta }\right)}_{i_{0}j}=0$ for all *j*. This statistic is asymptotically equivalent to the usual, likelihood ratio test statistic, and it is advantageous over the likelihood ratio test because it requires estimation of only one model (the restricted model). We make use of this advantage in the simulations, described later.

## 3. Tutorial

In this section, we demonstrate how the above tests can be carried out in R, using the package *lavaan* (Rosseel, 2012) for model estimation and *strucchange* (Zeileis et al., 2002; Zeileis, 2006) for testing. We use data from Froh et al. (2011) concerning the applicability of adult gratitude scales to youth, available in the R package *psychotools* (Zeileis et al., 2013). The data consist of responses to three adult gratitude scales from *n* = 1401 youth aged 10–19 years. The original authors were specifically interested in whether the scales were measurement invariant across age. Because the sample size at each age was unbalanced, the authors created age groups of approximately equal sample size. In the examples below, we test for measurement invariance across these age groups. For illustrative purposes, we conduct multiple tests and compare them to the traditional significance level of 0.05. In practice, however, one should generally adjust the significance level for the number of tests carried out. Additionally, because measurement invariance researchers often have large sample sizes, cross-validation methods can be useful to help verify the test results.

We focus on measurement invariance of the factor loadings associated with one of the scales in the dataset, the GQ-6 scale (McCullough et al., 2002). This scale consists of five Likert scale items (there is a sixth item that is omitted from analyses, following Froh et al.) with seven points each. We fit a one-factor model to these items, examining whether the factor model parameters are invariant with respect to age group. While the age group variable is best considered ordinal, for demonstration we consider its treatment as categorical, continuous, and ordinal. Each of these treatments is described below in a separate section.

### 3.1. Categorical treatment

Measurement invariance is most often tested using multiple groups models (see, e.g., van de Schoot et al., 2012). This amounts to assuming that our auxiliary variable is categorical (i.e., unordered), which is not true for the age groups in the data. However, we demonstrate the procedure for completeness.

To conduct the analysis, we first load the data and keep only complete cases for simplicity (though the tests can be applied to incomplete data).


*R> data("YouthGratitude", package = "psychotools")
R> compcases <- apply(YouthGratitude[, 4:28], 1,*
+                     *function(x) all(x %in% 1:9))
R> yg <- YouthGratitude[compcases, ]*


Next, we fit two models in *lavaan*: a one-factor model where loadings are restricted to be equal across age groups, and a one-factor model where loadings are free across age groups. This allows us to test a hypothesis of weak measurement invariance that was of interest to the original researchers (though, for ordinal variables, all types of measurement invariance can be examined via the tests described previously). By default, the code below sets the scale by fixing the first loading to 1.


*R> restr <- cfa("f1 =~ gq6_1 + gq6_2 + gq6_3 + gq6_4 +
+                      gq6_5",
+              data = yg, group = "agegroup",
+              meanstructure = TRUE,
+              group.equal = "loadings")
R> full <- cfa("f1 =~ gq6_1 + gq6_2 + gq6_3 + gq6_4 +
+                     gq6_5",
+              data = yg, group = "agegroup",
+              meanstructure = TRUE)*


Finally, we can get the results of a likelihood ratio test via the anova() function, which implies that the GQ-6 violates measurement invariance.


*R> anova(full, restr)*



Chi Square Difference Test



      Df   AIC   BIC Chisq Chisq diff Df diff Pr(>Chisq)
full  30 18947 19414   139
restr 50 18945 19308   177       38.1      20     0.0087


To obtain the asymptotically equivalent *LM*_*uo*_ (Equation 8), we can use the sctest() function from *strucchange*:


*R> sctest(restr, order.by = yg$agegroup, parm = 1:4,
+         vcov = "info", functional = "LMuo")*



      M-fluctuation test



data:  restr
f(efp) = 31.4, p-value = 0.05018


This command specifies that we assess the parameters 1–4 of model restr after ordering the observations according to agegroup. Additionally, the observed information matrix is used as the variance-covariance matrix. Note that the model parameters 1–4 are the factor loadings supplied by *lavaan*, which can be seen by inspecting coef(restr). This also leads to somewhat smaller test statistics that are very close to being significant at the 5% level.

Because our sample size is large, the likelihood ratio test is known to be sensitive to small measurement invariance violations (Bentler and Bonett, 1980). That is, the LRT and *LM*_*uo*_ test from Equation (8) are sensitive to small measurement invariance violations that are not likely to be of interest to researchers. For example, imagine that the 15-year-olds' parameters are slightly different than the other age groups. The 15-year-olds are in the middle of the age groups, and there is not likely to be any theoretical justification for 15-year-olds differing from every other age group. One solution to this problem would be the Bayesian, approximate invariance methods described in the introduction (Muthén and Asparouhov, 2013). Alternatively, we can use the “ordinal” score-based statistics (from Equations (6), (7)) to obtain tests that are sensitive to the ordering of age.

### 3.2. Continuous treatment

If we are interested in measurement invariance violations that are monotonic with the age groups, it is perhaps simplest to treat the age groups as continuous. In doing so, we can use the statistics from Equations (3–5). That is, we can fit a model whose parameters are restricted to be equal across all individuals and then examine how individuals' scores *s*($\hat{\theta }$; *x*_*i*_) fluctuate with their age (where age ties are broken arbitrarily, using the original order of the observations within each age group). This is demonstrated below, with similar code being useful when one is testing for measurement invariance w.r.t. truly continuous variables.

Again, we employ the sctest() function to assess parameters 1–4 from the restricted model restr after ordering w.r.t. agegroup:


*R> dm <- sctest(restr, order.by = yg$agegroup,
+               parm = 1:4, vcov = "info",
+               functional = "DM")
R> cvm <- sctest(restr, order.by = yg$agegroup,
+               parm = 1:4, vcov = "info",
+               functional = "CvM")
R> maxlm <- sctest(restr, order.by = yg$agegroup,
+               parm = 1:4, vcov = "info",
+               functional = "maxLM")
R> c(dm$p.value, cvm$p.value, maxlm$p.value)*



[1] 0.03804 0.11557 0.00414


We see that two of the three *p*-values output at the end of the code are larger than that associated with the LRT (with the CvM statistic being non-significant).

The tests carried out here assume a unique ordering of individuals by age, but this is obviously not the case. To compute the statistics and *p*-values, the *strucchange* package implicitly employed the (arbitrary) ordering of individuals who are tied on age. If we were to change this ordering, the resulting statistics and *p*-values would also change, potentially switching significant results to being non-significant and vice versa. Clearly, this is problematic. To accurately account for the multiple observations at the same age level, we must use the ordinal tests from Equations (6) and (7). These are described next.

### 3.3. Ordinal treatment

The main difference between the ordinal test statistics and their continuous counterparts is that the ordinal statistics are unchanged when re-ordering individuals within the same age group. To compute the test statistics, we allow the scores of all tied individuals to enter the cumulative sum (Equation (2)) simultaneously. This results in modified critical values and test statistics that are sensitive to measurement invariance violations that are monotonic w.r.t. age group.

To carry out the tests, we can rely on the same function that we used for the continuous test statistics. As mentioned previously, calculation of the max *LM*_*o*_ statistic (Equation (7)) can be lengthy from the need to simulate critical values (though see the end of this section, which provides a partial speed-up).


*R> wdmo <- sctest(restr, order.by = yg$agegroup,
+                 parm = 1:4, vcov = "info",
+                 functional = "WDMo")
R> maxlmo <- sctest(restr, order.by = yg$agegroup,
+                 parm = 1:4, vcov = "info",
+                 functional = "maxLMo")*



*R> c(wdmo$p.value, maxlmo$p.value)*



[1] 0.0588 0.0970


In computing the ordinal test statistics, we obtain *p* = 0.059 and *p* = 0.097, respectively.^1^ Both *p*-values are clearly larger than that of the likelihood ratio test and neither is significant at α = 0.05. This provides evidence that there is no measurement invariance violation that is monotonic with age group. Instead, given the large sample size, the likelihood ratio test may be overly sensitive to anomalous, non-monotonic violations at one (or a few) age groups.

**Figure 1:**
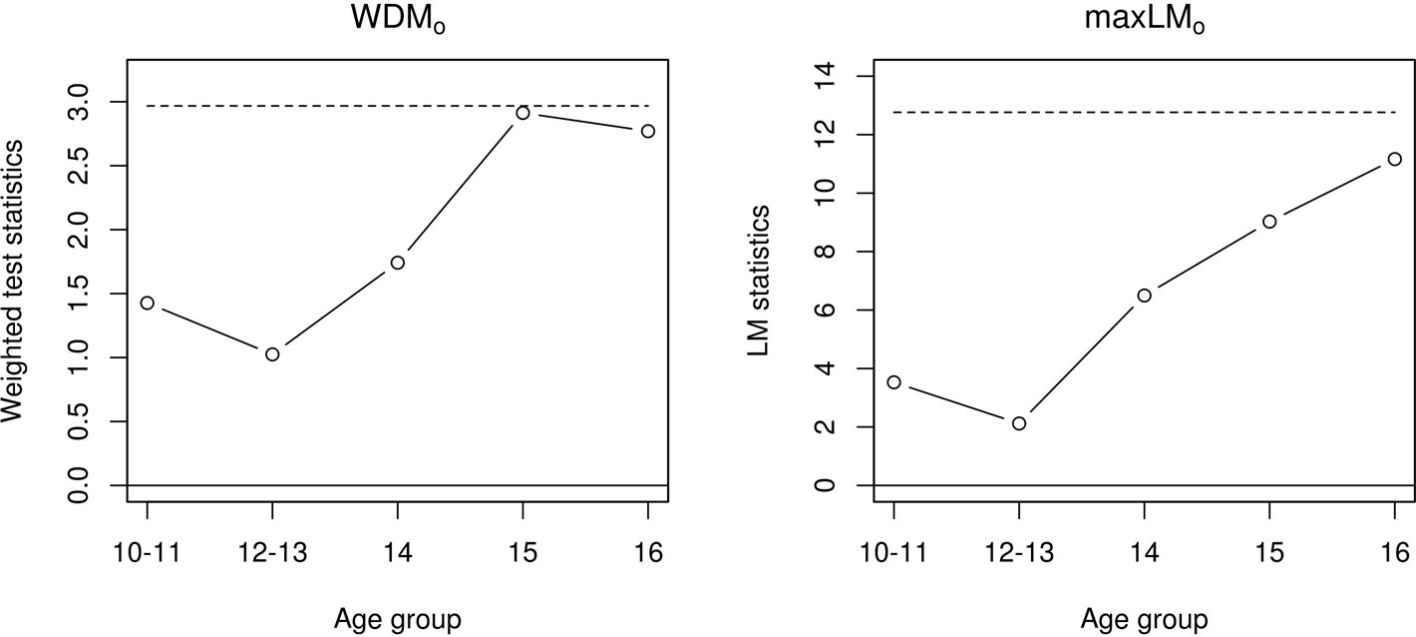
**Fluctuation processes for the *WDM*_*o*_ statistic (left panel) and the max *LM*_*o*_ statistic (right panel)**.

In addition to test statistics, “instability plots” can be generated by setting plot = TRUE in the sctest() calls above. Figure 1 displays the resulting plots, which represent the ordinal statistics' fluctuations across levels of age group. The *x*-axis reflects age group and the *y*-axis reflects test statistic values (larger values reflect more instability), with the dashed horizontal lines reflecting critical values. The hypothesis of measurement invariance is rejected if the sequence of test statistics crosses the critical value. While the measurement invariance tests are non-significant, the plots imply some instability in the older age groups (15, 16).

Finally, if the user anticipates multiple calculations of the max *LM*_*o*_ statistic for a specific dataset, it is possible to save time by simulating critical values once and re-using them for multiple tests. We can use the ordL2BB() function to generate critical values and store them in an object mLMo, say. Then, this object can be employed to obtain the test statistic in the usual manner.


*R> mLMo <- ordL2BB(yg$agegroup)
R> maxlmo <- sctest(restr, order.by = yg$agegroup,
+                   parm = 1:4, vcov = "info",
+                   functional = mLMo)*


The ordL2BB() command automatically generates critical values for testing 1–20 parameters at a time. If only a smaller number of parameters (e.g., only up to 6) is to be tested, some computation time can be saved by setting the nproc argument accordingly (e.g., nproc = 1:6). In the same way, nproc can be employed to simulate higher-dimensional fluctuation processes suitable for testing more parameters. One can re-use mLMo in this manner for further tests of the youth gratitude data. Critical values must be resimulated for new data, however, because they depend on the proportion of individuals observed at each level of the ordinal variable (denoted *t*_ℓ_ for Equation (7)).

In the above sections, we have illustrated the score-based tests' computation in R. We suspect that the ordinal tests will be most popular with users, because measurement invariance tests are typically carried out across categories (ordered or not), as opposed to continuous variables. Thus, in the sections below, we conduct novel simulations to study the ordinal statistics' expected behavior in practice. In particular, we wish to study (1) the extent to which the ordinal statistics attribute measurement invariance violations to the correct parameter(s), and (2) the extent to which the tests are robust to model misspecification. These issues are especially important to examine because SEMs are typically complex, with many inter-related parameters that may exhibit measurement invariance. Previous applications of score-based tests have typically focused on regression-like models with only a small number of parameters that may exhibit instability (e.g., Zeileis and Hornik, 2007). Thus, the simulations here provide general evidence about the extent to which the tests accurately capture instabilities in complex models.

## 4. Simulation 1

In Simulation 1, we examined the extent to which the proposed tests can “localize” a measurement invariance violation. If, say, a factor loading violates measurement invariance, it is plausible that this violation impacts other parameter estimates, including factor covariances, intercepts or the unique variance associated with the manifest variable in question. Thus, the goal of the Simulation 1 is to examine the extent to which the proposed tests attribute the measurement invariance violation to the parameters that are truly in violation.

### 4.1. Methods

**Figure 2:**
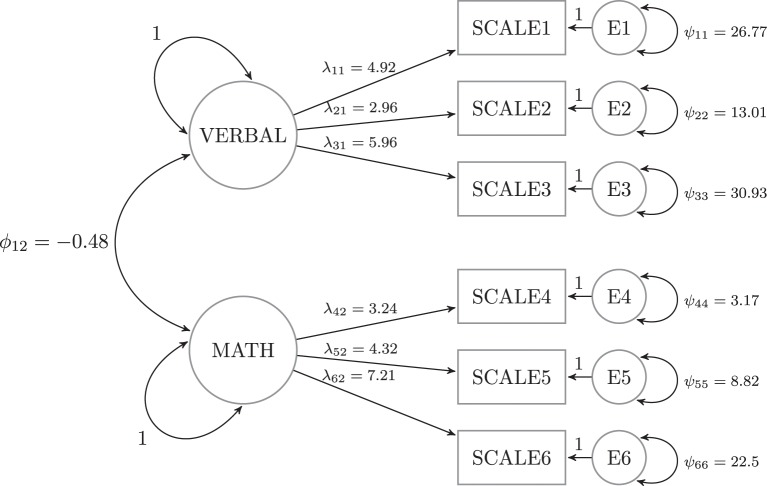
**General model used for the simulations**.

To examine these issues, we generated data from a two-factor model with three indicators each (see Figure 2). The measurement invariance violation occurred in one of four places: the factor loading associated with Scale 1 (λ_11_), the intercept (μ_11_), the unique variance (ψ_11_), or the factor covariance (ϕ_12_). Note that the latter violation is not necessarily a measurement invariance (e.g., Meredith, 1993), but it is still a parameter instability that can occur in this type of model. We then tested for measurement invariance (parameter instability) in seven subsets of parameters: each of the four individual parameters noted above, all six factor loadings, all six unique variances and all six intercepts.

Power and Type I error were examined across three sample sizes (*n* = 120, 480, 960), three numbers of categories (*m* = 4,8,12), and 17 magnitudes of invariance violations (described in the following sentences). The measurement invariance violations began at level 1 + *m*/2 of the auxiliary variable *V* and were consistent thereafter: individuals below level 1 + *m*/2 of *V* deviated from individuals at or above level 1 + *m*/2 by *d* times the parameters' asymptotic standard errors (scaled by $\sqrt{n}$), with *d* = 0, 0.25, 0.5, …, 4 (see replication code for specific values of the standard errors). For each combination of sample size (*n*) × violation magnitude (*d*) × violating parameter × categories (*m*), 5000 datasets were generated and tested. Statistics from Equations (6–8) were examined. As mentioned previously, Equation (8) is asymptotically equivalent to the usual likelihood ratio test. Thus, this statistic provides information about the relative performance of the ordinal statistics vs. the LRT.

In all conditions, we maintained equal sample sizes in each subgroup of the ordinal variable. Aside from the parameter changes that reflect measurement invariance, the fitted models matched the data generating model.

### 4.2. Results

**Figure 3:**
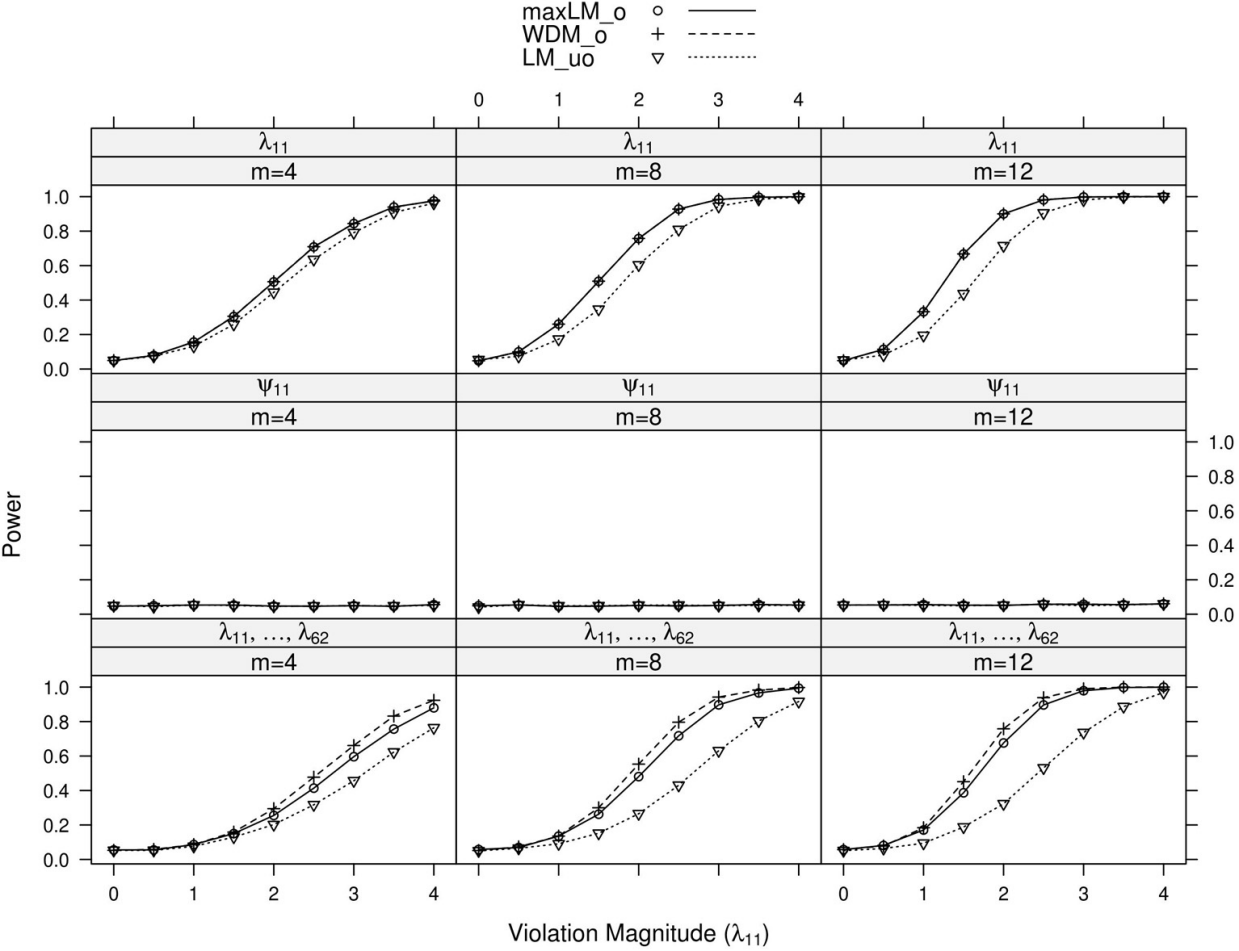
**Simulated power curves for max *LM*_*o*_, *WDM*_*o*_, and *LM*_*uo*_ across three levels of the ordinal variable *m* and measurement invariance violations of 0–4 standard errors (scaled by $\sqrt{n}$), Simulation 1**. The parameter violating measurement invariance is λ_11_. Panel labels denote the parameter(s) being tested and the number of levels of the ordinal variable *m*.

**Figure 4:**
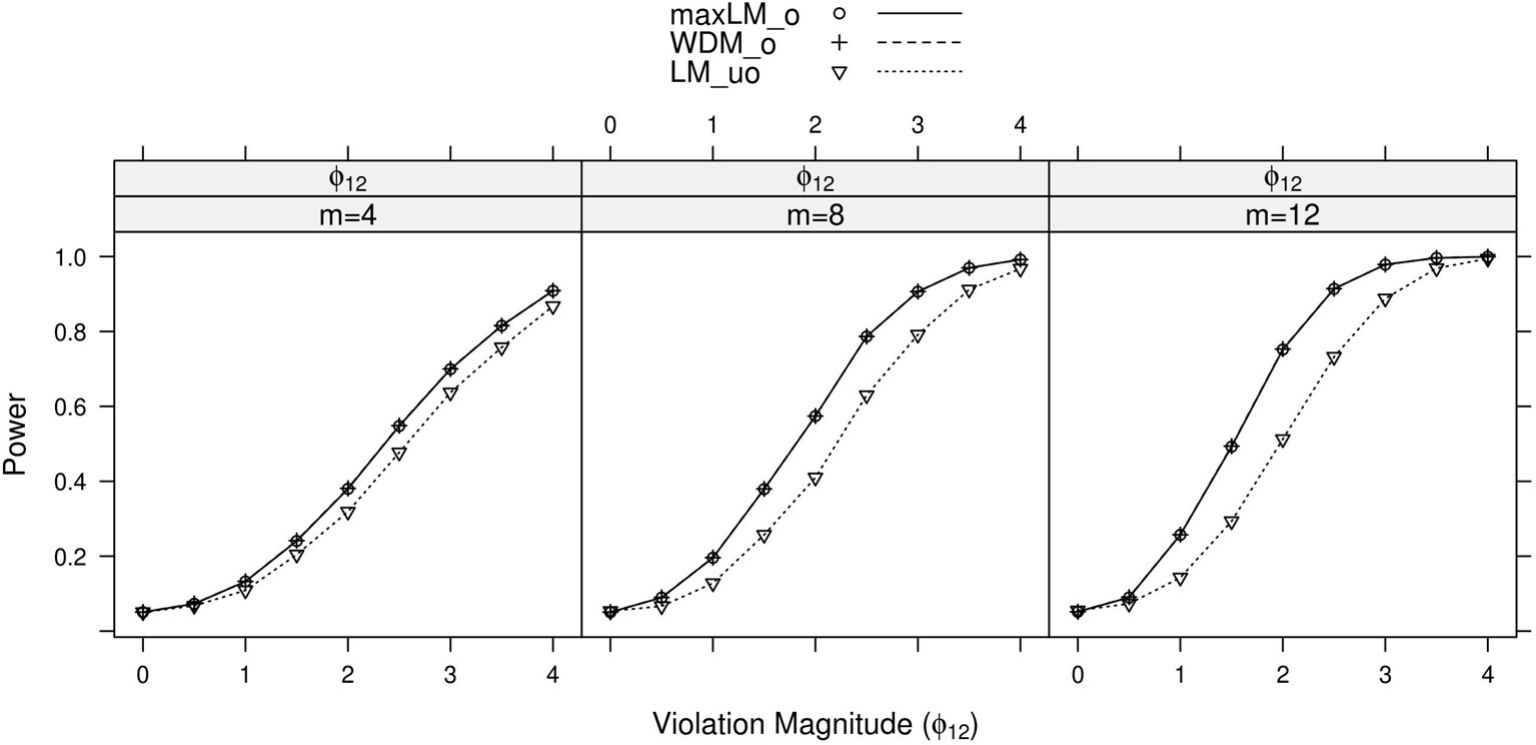
**Simulated power curves for max *LM*_*o*_, *WDM*_*o*_, and *LM*_*uo*_ across three levels of the ordinal variable *m*, and measurement invariance violations of 0–4 standard errors (scaled by $\sqrt{n}$), Simulation 1**. The parameter violating measurement invariance is ϕ_12_. Panel labels denote the parameter(s) being tested and the number of levels of the ordinal variable *m*.

**Figure 5:**
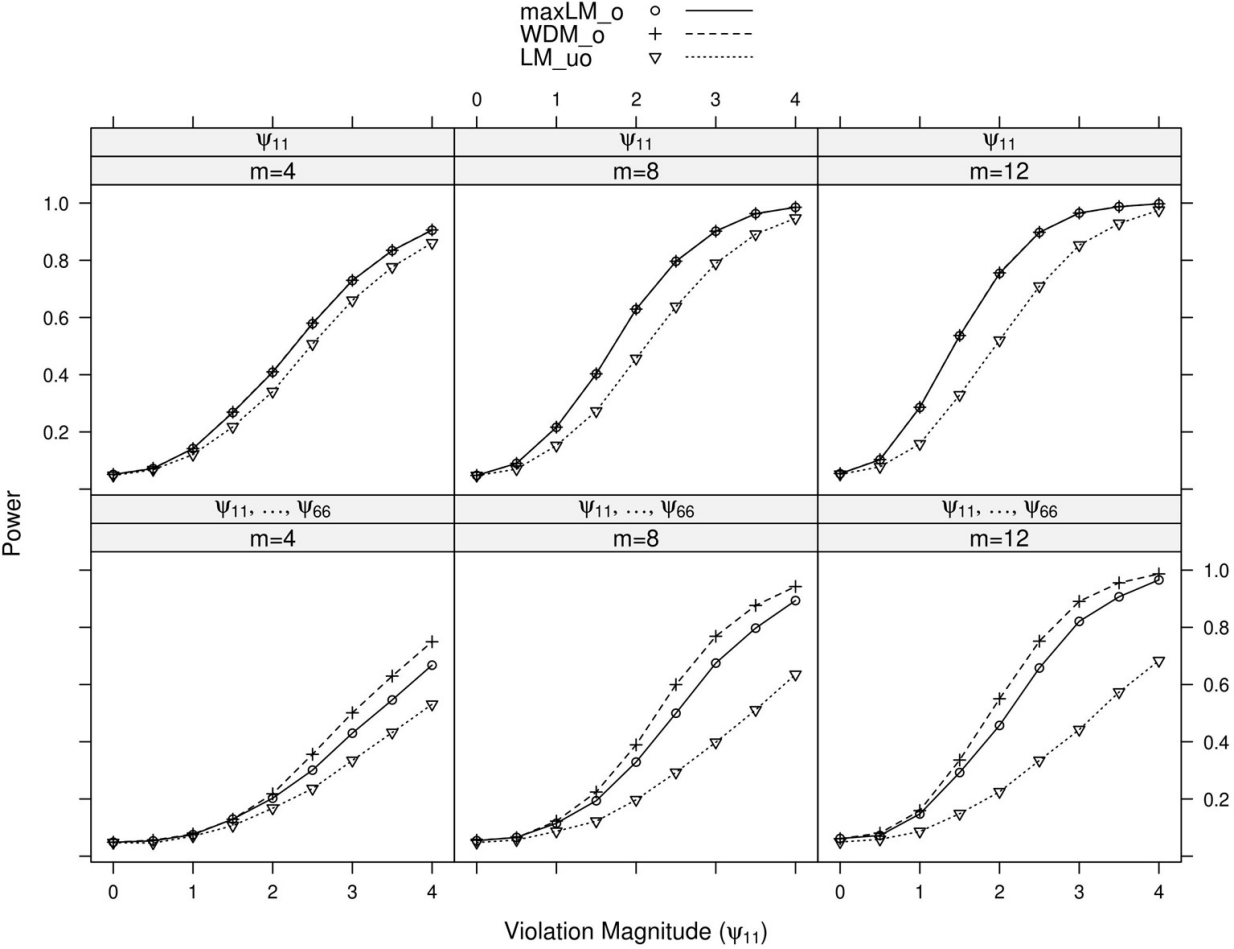
**Simulated power curves for max *LM*_*o*_, *WDM*_*o*_, and *LM*_*uo*_ across three levels of the ordinal variable *m* and measurement invariance violations of 0–4 standard errors (scaled by $\sqrt{n}$), Simulation 1.** The parameter violating measurement invariance is ψ_11_. Panel labels denote the parameter(s) being tested and the number of levels of the ordinal variable *m*.

**Figure 6:**
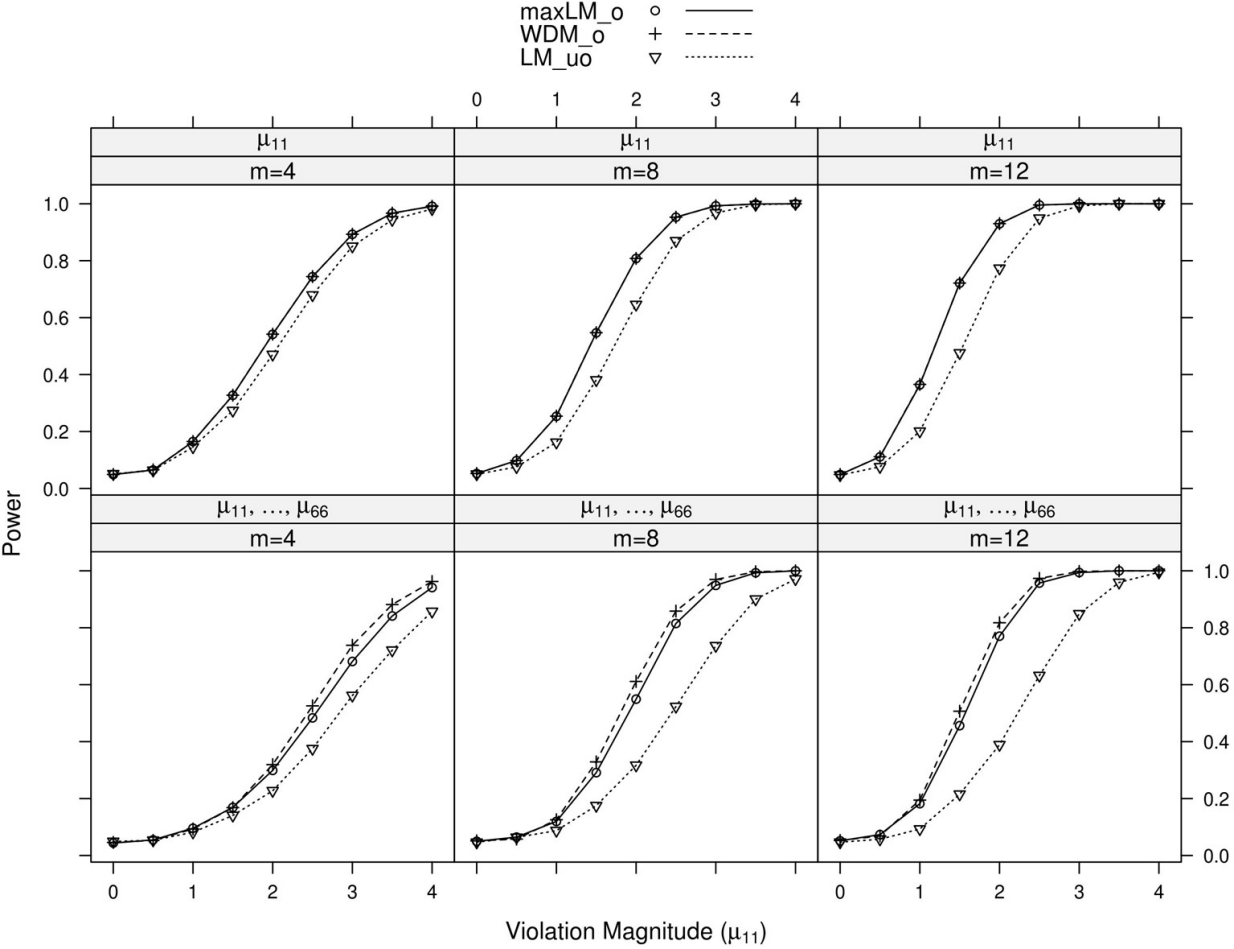
**Simulated power curves for max *LM*_*o*_, *WDM*_*o*_, and *LM*_*uo*_ across three levels of the ordinal variable *m* and measurement invariance violations of 0–4 standard errors (scaled by $\sqrt{n}$), Simulation 1.** The parameter violating measurement invariance is μ_11_. Panel labels denote the parameter(s) being tested and the number of levels of the ordinal variable *m*.

Full simulation results are presented in Figures 3–6. Figure 3 displays power curves as a function of violation magnitude in the factor loading λ_11_, with the parameters being tested changing across rows, the number of levels *m* of the ordinal variable *V* across columns, and lines reflecting different test statistics. Figures 4–6 display similar power curves when the factor covariance ϕ_12_, error variance ϵ_11_, and intercept μ_11_ violate measurement invariance, respectively. In these figures, we generally show tests associated with parameters that exhibited non-zero power curves. For example, in Figure 3, the middle row shows that power for tests of ψ_11_ stays near zero for all values of *m* and *d*. Similar rows have been omitted from this figure and other figures.

Within each panel of Figures 3–6, the three lines reflect the three test statistics. It is seen that the two ordinal statistics exhibit similar results, with max *LM*_*uo*_ demonstrating lower power across all situations. This demonstrates the sensitivity of the ordinal statistics to invariance violations that are monotonic with *V*. In situations where only one parameter is tested, *WDM*_*o*_ and max *LM*_*o*_ exhibit equivalent power curves. This is because, when only one parameter is tested, the statistics are equivalent.

From these figures, one generally observes that the tests isolate the parameter violating measurement invariance. Additionally, the tests have somewhat higher power to detect measurement invariance violations in the factor loading, factor covariance, and intercept parameters, as opposed to the error variance parameter. Finally, simultaneous tests of all factor loadings, all intercepts, or all error parameters result in decreased power, as compared to the situation where one tests only the violating parameter. This occurs because, in testing a subset of parameters (only one of which violates measurement invariance), we are dampening the signal of a measurement invariance violation. This “dampening” effect is more apparent for the max *LM*_*o*_ statistic, because it involves a sum across all tested parameters (see Equation 7). Conversely, *WDM*_*o*_ takes the maximum over parameters (Equation 6), so that invariant parameters have no impact on this statistic.

In summary, we found that the proposed tests can attribute measurement invariance violations to the correct parameter. This provides evidence that, in practice, one can have confidence in the tests' abilities to locate the measurement invariance violation. Of course, this statement is qualified by the fact that, in this simulation, the model was correctly specified. In the following simulation, we examine the tests' performance in the likely situation of model misspecification.

## 5. Simulation 2

In Simulation 2, we examine the extent to which the results of Simulation 1 are robust to model misspecification. Specifically, we generate data from the factor analysis model used in the previous section, except that the model contains an extra loading from the second factor to Scale 1. The estimated model matches that displayed in Figure 2, however, resulting in model misspecification. The goal of this simulation is to examine the proposed statistics' power to detect measurement invariance violations (and to attribute the violation to the correct parameter) under this misspecification.

### 5.1. Method

A measurement invariance violation could occur in each of the four parameters from Simulation 1 (factor loading, factor covariance, unique variance, and intercept), and a violation could also occur in the extra, unmodeled loading. In each condition, a single parameter exhibited the violation. Sample size and magnitude of measurement invariance violation were manipulated in the same way as they were in Simulation 1. The tested parameters were also the same as Simulation 1.

### 5.2. Results

**Figure 7:**
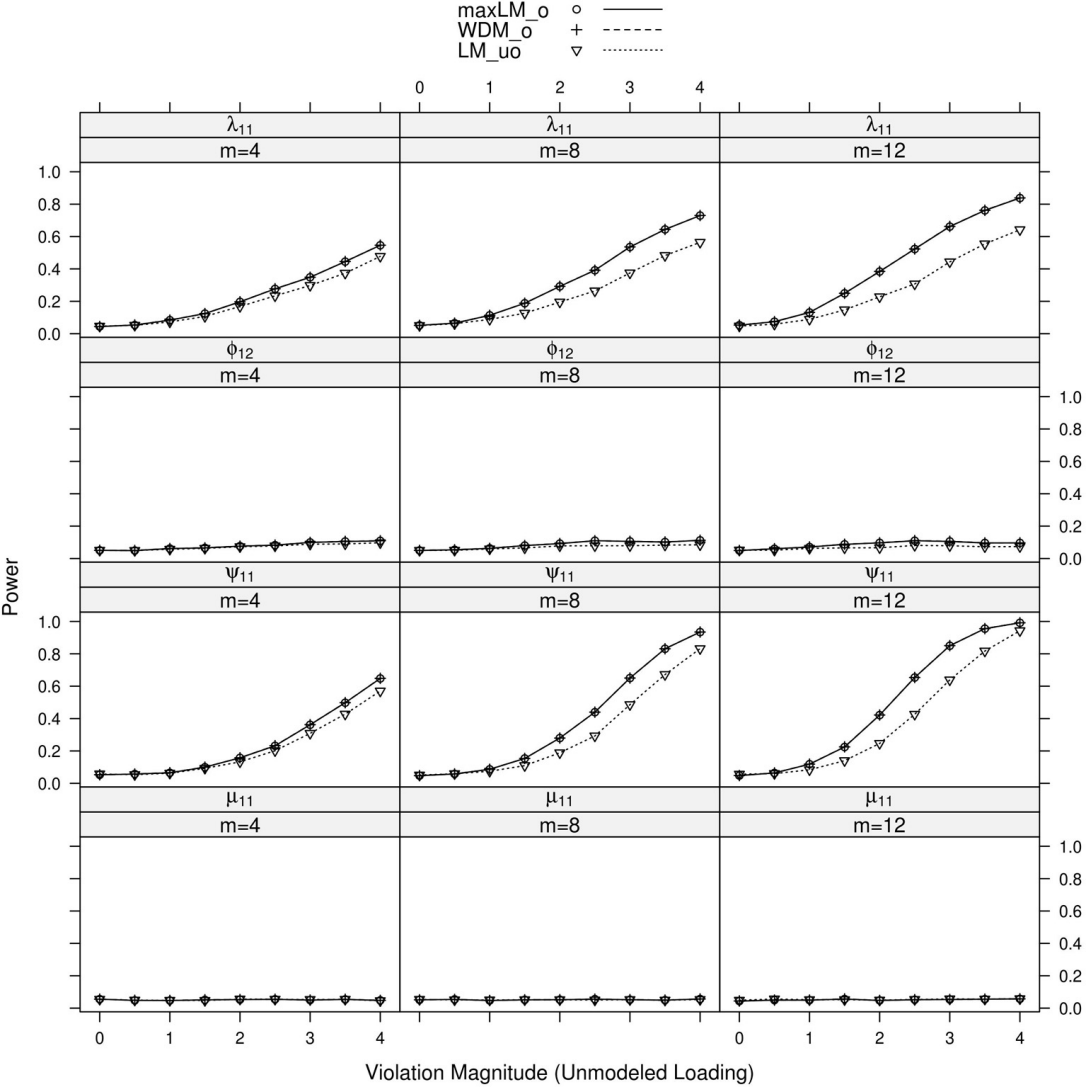
**Simulated power curves for max *LM*_*o*_, *WDM*_*o*_, and *LM*_*uo*_ across three levels of the ordinal variable *m* and measurement invariance violations of 0–4 standard errors (scaled by $\sqrt{n}$), Simulation 2.** The parameter violating measurement invariance is the unmodeled loading. Panel labels denote the parameter(s) being tested and the number of levels of the ordinal variable *m*.

Results of primary interest are conditions where the unmodeled loading violates measurement invariance. A subset of results is displayed in Figure 7. One can generally observe that tests of the first loading and unique variance exhibited high “power,” which is actually a high Type I error rate here. This Type I error is also observed when testing all loadings and all unique variances (see the Supplementary Material). Tests associated with the factor covariance and intercept did not demonstrate this error, however. In terms of specific statistic performance, max *LM*_*o*_ and *WDM*_*o*_ demonstrated higher Type I error than *LM*_*uo*_ in each panel, especially with increasing levels. This is likely because the unmodeled loading's non-invariance was monotonic with *V*; if it were not monotonic, we would expect *LM*_*uo*_ to have higher Type I error.

When the parameter violating measurement invariance was modeled, results were generally the same as Simulation 1. When the modeled factor loading, λ_11_, violated measurement invariance, the statistics were generally able to pick up the violation despite the misspecification. Similar results were observed when the unique variance, intercept and factor covariance parameters violated measurement invariance; these results are all shown in the Supplementary Material. In particular, power of the ordered statistics was higher than power of the unordered statistic in each panel.

In summary, the proposed test statistics appear robust to unmodeled loading parameters, when the unmodeled loading does not violate measurement invariance and when the rest of the model is correctly specified (save for the measurement invariance violation). If the unmodeled loading does violate measurement invariance, the tests can still detect measurement invariance violations. The violations are assigned to modeled parameters that do not violate measurement invariance, however. The impacted parameters include the error variance and other loadings associated with the manifest variable that has an unmodeled loading. Thus, as for other tests of measurement invariance, it is important to study the extent to which the hypothesized model includes all parameters of importance (i.e., the extent to which the model is well specified).

One could begin to study model misspecification by fitting models with different discrepancy functions (say, a multivariate normal function and a generalized least squares function). If parameter estimates differ greatly across the functions, then this implies model misspecification. Additionally, if one has a large sample size, one could split the data into subgroups and examine consistency of results across subgroups. These issues are important for all the tests discussed here (score-based or otherwise).

## 6. General discussion

In this paper, we first described a novel family of test statistics for measurement invariance and illustrated their use via the R packages *lavaan* and *strucchange*. Next, we examined these statistics' abilities to identify the parameter violating measurement invariance under well-specified and misspecified models. We found that the proposed statistics could generally isolate the model parameter violating measurement invariance, so long as the violating parameter is included in the model.

In the remainder of the paper, we first compare the use these tests to the use of traditional tests in practice. We then discuss test extension to other fit functions and to other specialized models.

### 6.1. Applications

Many of the applications in this volume, along with many measurement invariance applications in general, focus on testing across unordered categories such as nations or gender. As discussed earlier in this paper, the score-based tests for unordered categories are equivalent to the usual likelihood ratio test. Given a measurement invariance violation across these unordered categories, however, researchers typically wish to know why the violation occurred. At this point, researchers may examine education level, socioeconomic status, income levels, and so on across the unordered categories. These variables are often ordinal or continuous in nature, so that the family of tests described in this paper are applicable. This is a first step toward describing why measurement invariance violations occur, as opposed to simply detecting measurement invariance violations. The tests described here are convenient for this purpose, as they do not require a new model to be estimated for each ordinal variable. Instead, each ordinal variable defines an ordering of observations, which in turn yields a test statistic that is specific to that ordinal variable.

### 6.2. Extension

In this paper, we focused on testing for measurement invariance in factor analysis models that assume multivariate normality and that are estimated via maximum likelihood (ML). The family of tests described here generally apply to estimation methods that maximize/minimize a fit function, however (see Zeileis and Hornik, 2007), so they are potentially applicable to alternative SEM discrepancy functions such as generalized least squares (e.g., Browne and Arminger, 1995). Score calculation for these alternative discrepancy functions has not been implemented (to our knowledge), though the calculation could be implemented. Test statistic calculation and inference would then proceed in exactly the same manner as the calculation and inference illustrated in this paper. Study of the proposed tests' application to larger SEMs is warranted.

In addition to alternative fit functions, the tests can be extended to other models estimated via ML. Of primary relevance to the topic of measurement invariance, the tests can be extended to item response models to examine differential item functioning. In particular, Strobl et al. (2014) studied application of these tests to the Rasch model, using them as the basis of a recursive partitioning procedure that segments subgroups of individuals who exhibit DIF. Further study and extension of these tests for IRT are warranted.

## Computational details

All results were obtained using the R system for statistical computing (R Core Team, 2013), version 3.1.0, employing the add-on package *lavaan* 0.5–16 (Rosseel, 2012) for fitting of the factor analysis models and *strucchange* 1.5–0 (Zeileis et al., 2002; Zeileis, 2006) for evaluating the parameter instability tests. R and both packages are freely available under the General Public License 2 from the Comprehensive R Archive Network at http://CRAN.R-project.org/. R code for replication of our results is available at http://semtools.R-Forge.R-project.org/ and also in an online supplement to this article.

### Conflict of interest statement

The authors declare that the research was conducted in the absence of any commercial or financial relationships that could be construed as a potential conflict of interest.

## Footnotes

^1^To replicate both *p*-values exactly, R's random seed needs to be set by set.seed(1090) prior to each sctest() call.

## Supplementary material

The accompanying zip file contains R code for replication of all analyses and simulations from the article. File descriptions appear below.

- mz-frontiers.R: Model estimation functions for simulations.
- sim-frontiers.R: Functions for data generation, power evaluation, and power summaries.
- replication-frontiers.R: Code for the tutorial and simulations, utilizing the other two files.
