# Supplementary material for: Score-based tests of measurement invariance: use in practice
Source: Front Psychol. 2014 May 30;5:438. doi: 10.3389/fpsyg.2014.00438 (PMC4038958; doi:10.3389/fpsyg.2014.00438)

Supplementary Material to Wang, Merkle, & Zeileis

The Supplementary Material contains additional results from Simulation 2, where there existed model misspecification (lacking one loading from Scale 1 to Math). Figures 1 to 5 display results when the unmodeled loading, the loading λ_{11}, the covariance ϕ_{12}, the error term ψ_{11}, and the intercept μ_{11} violate invariance, respectively. Results are only shown for simulation conditions exhibiting power curves that increased from zero. These results were generally the same as the Simulation 1 results.

Figure 1. Simulated power curves for $\max LM_o$, WDM_o, and LM_{uo} across three levels of the ordinal variable m and measurement invariance violations of 0–4 standard errors (scaled by $\sqrt{n}$), Simulation 2. The parameter violating measurement invariance is the unmodeled loading. Panel labels denote the parameter(s) being tested and the number of levels of the ordinal variable m.

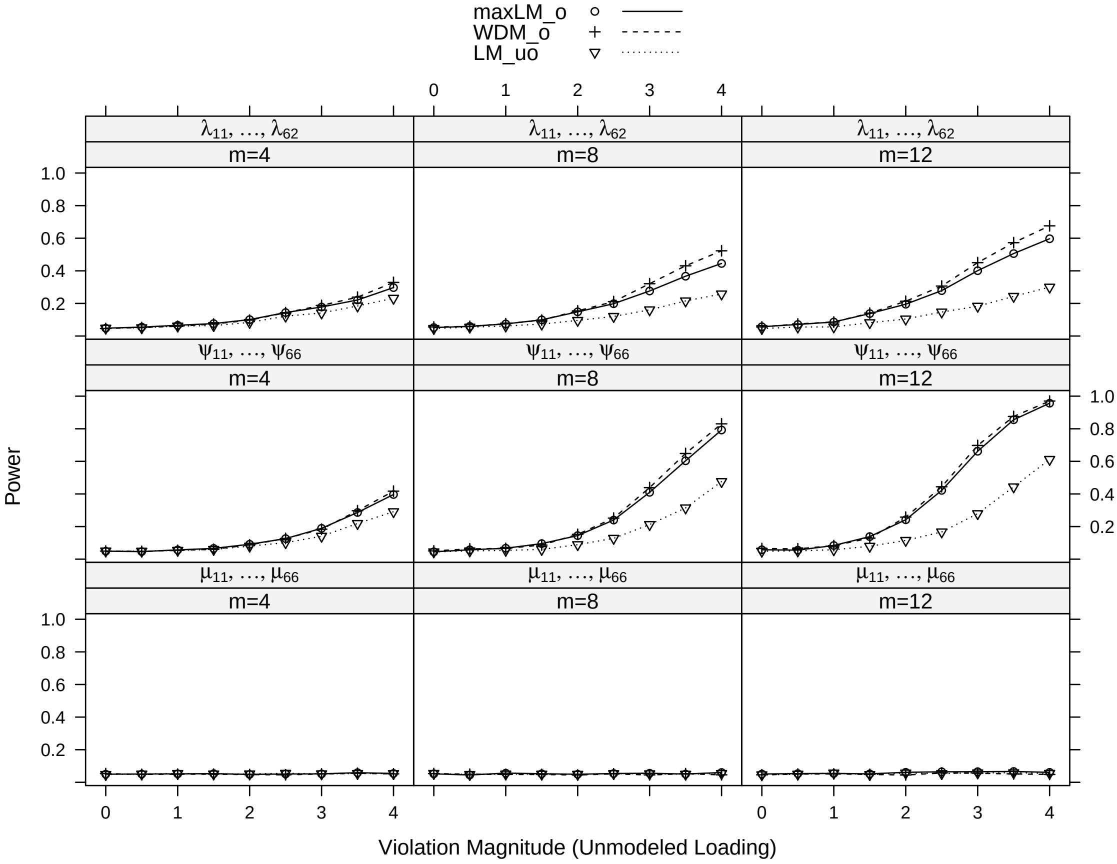

Figure 2. Simulated power curves for $\max LM_o$, WDM_o, and LM_{uo} across three levels of the ordinal variable m and measurement invariance violations of 0–4 standard errors (scaled by $\sqrt{n}$), Simulation 2. The parameter violating measurement invariance is λ_{11}. Panel labels denote the parameter(s) being tested and the number of levels of the ordinal variable m.

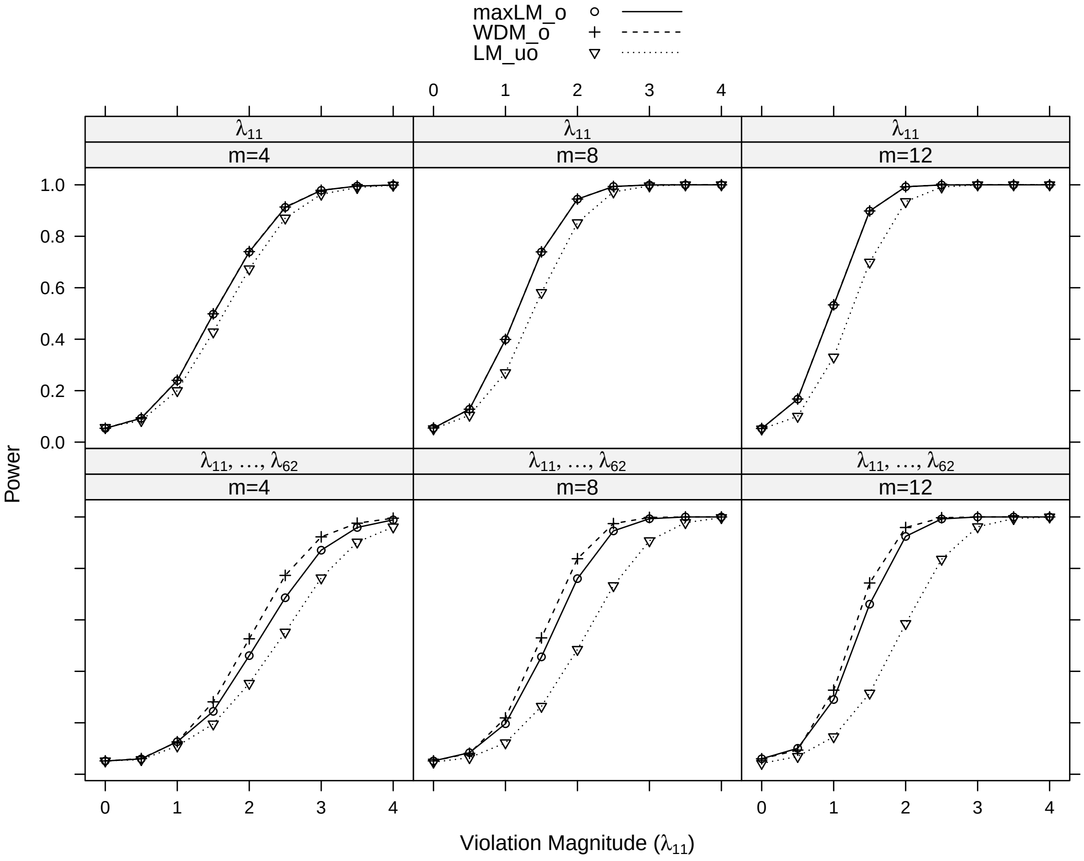

Figure 3. Simulated power curves for $\max LM_o$, WDM_o, and LM_{uo} across three levels of the ordinal variable m and measurement invariance violations of 0–4 standard errors (scaled by $\sqrt{n}$), Simulation 2. The parameter violating measurement invariance is ϕ_{12}. Panel labels denote the parameter being tested and the number of levels of the ordinal variable m.

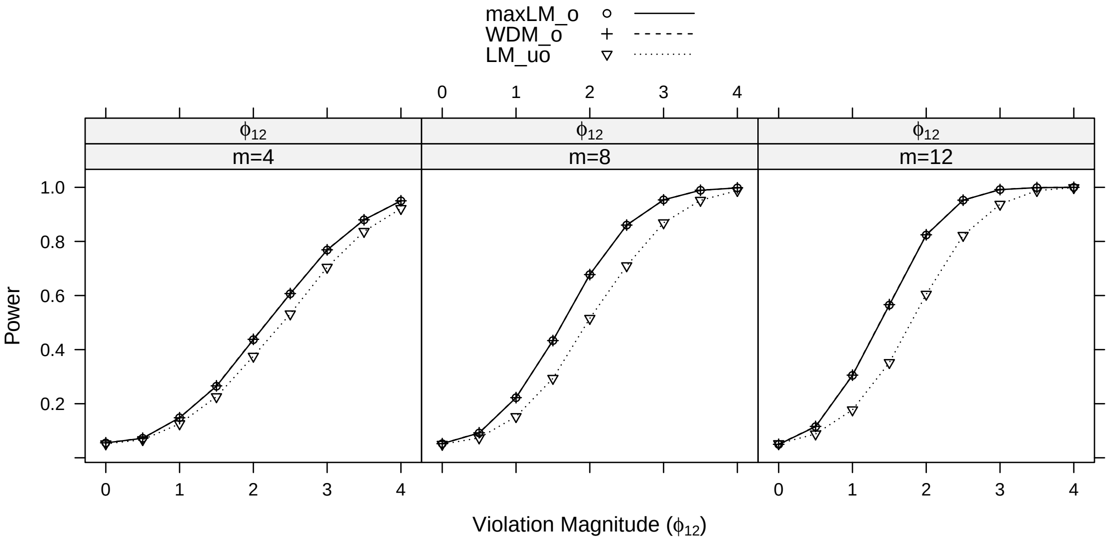

Figure 4. Simulated power curves for $\max LM_o$, WDM_o, and LM_{uo} across three levels of the ordinal variable m and measurement invariance violations of 0–4 standard errors (scaled by $\sqrt{n}$), Simulation 2. The parameter violating measurement invariance is ψ_{11}. Panel labels denote the parameter(s) being tested and the number of levels of the ordinal variable m.

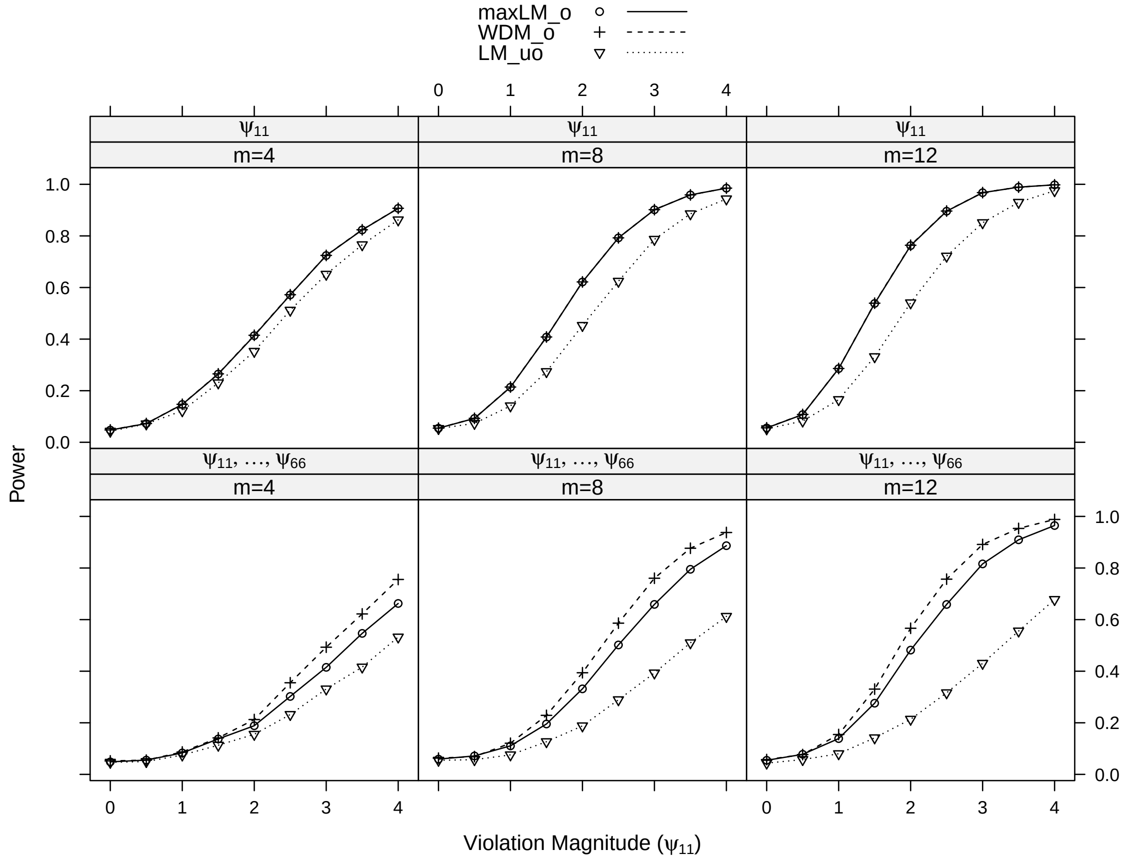

Figure 5. Simulated power curves for $\max LM_o$, WDM_o, and LM_{uo} across three levels of the ordinal variable m and measurement invariance violations of 0–4 standard errors (scaled by $\sqrt{n}$), Simulation 2. The parameter violating measurement invariance is μ_{11}. Panel labels denote the parameter(s) being tested and the number of levels of the ordinal variable m.

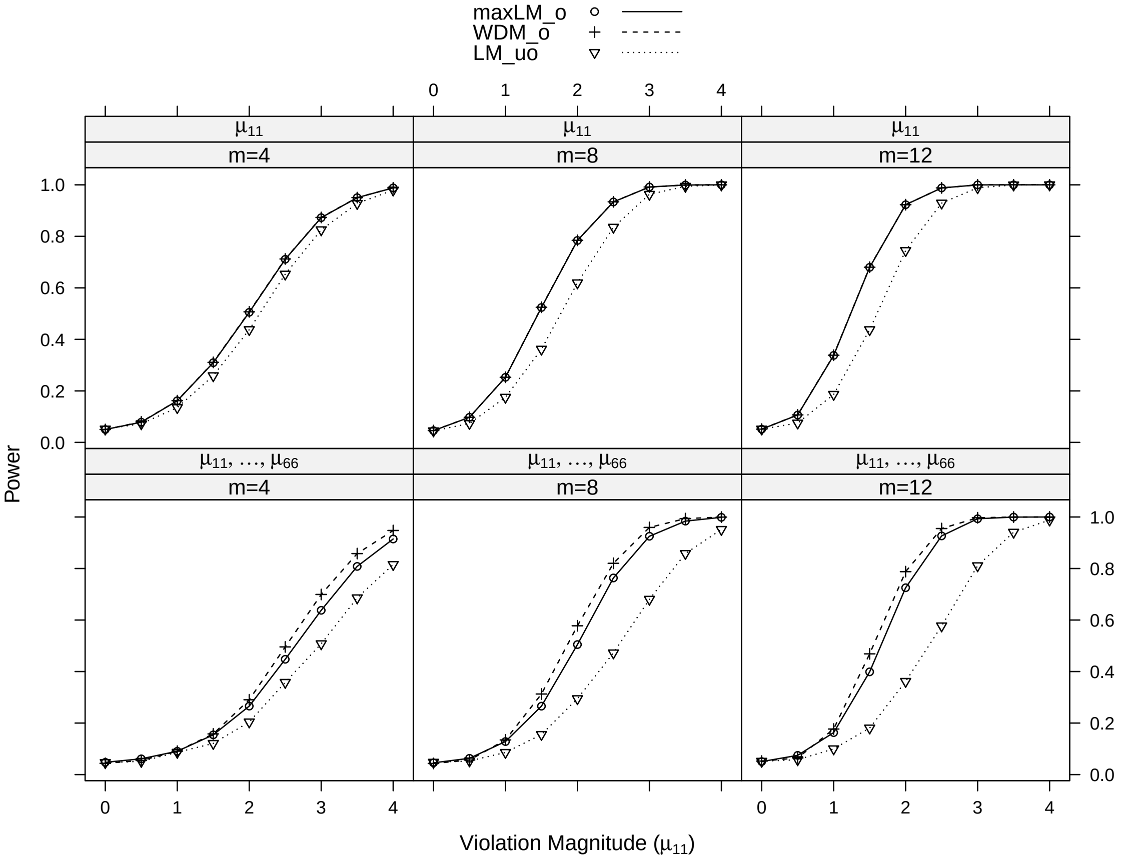

Supplement: Supplementary file 1 [file Presentation1.PDF]
